# Fatty acid supplementation into warming solutions improves pregnancy outcomes after single vitrified‐warmed cleavage stage embryo transfers

**DOI:** 10.1002/rmb2.12517

**Published:** 2023-05-08

**Authors:** Ayumi Amagai, Kenji Ezoe, Tetsuya Miki, Kiyoe Shimazaki, Tadashi Okimura, Keiichi Kato

**Affiliations:** ^1^ Kato Ladies Clinic Tokyo Japan

**Keywords:** embryo transfer, embryonic development, fatty acid supplementation, morphokinetics, pregnancy

## Abstract

**Purpose:**

This study aimed to examine the embryonic development of human 4‐cell stage embryos after warming with fatty acids (FAs) and to assess the pregnancy outcomes after single vitrified‐warmed cleavage stage embryo transfers (SVCTs).

**Methods:**

Experimental study: A total of 217 discarded, vitrified human 4‐cell stage embryos donated for research by consenting couples were used. The embryos were warmed using the fatty acid (FA)‐supplemented solutions (FA group) or nonsupplemented solutions (control group). The developmental rate, morphokinetics, and outgrowth competence were analyzed. Clinical study: The treatment records of women undergoing SVCT in natural cycles between April and September 2022 were retrospectively analyzed (April–June 2022, control group; July–September 2022, FA group).

**Results:**

Experimental study: The rate of morphologically good blastocysts was significantly higher in the FA group than in the control group (*p* = 0.0302). The morphokinetics during cleavage, morula, and blastocyst stages were comparable between the groups. The outgrowth was significantly increased in the FA group (*p* = 0.0438). Clinical study: The rates of implantation, clinical pregnancy, and ongoing pregnancy after SVCTs were significantly increased in the FA group (*p* = 0.0223–0.0281).

**Conclusions:**

Fatty acid‐supplemented warming solutions effectively improve embryo development to the blastocyst stage and pregnancy outcomes after SVCTs.

## 1 INTRODUCTION

The cycle number of frozen embryo transfers (FETs) has progressively increased due to the establishment of vitrification techniques and freeze‐all strategy.^1^, ^2^, ^3^ In fact, 89.4% of births derived from assisted reproductive technologies are obtained from FET cycles in Japan.^3^ The cryopreservation technique enables embryos to be transferred at the optimal time without the detrimental effects of ovarian stimulation on endometrial function; therefore, improved pregnancy outcomes are expected after FETs compared with those after fresh embryo transfers.^4^, ^5^, ^6^ Furthermore, recent studies reported that maternal and perinatal complications could be reduced by the FETs in the natural cycle^7^ but not in the hormone replacement cycle.^8^ In addition, since the use of preimplantation genetic testing (PGT) has been broadly increasing,^9^, ^10^, ^11^, ^12^, ^13^ the demand for FETs is expected to continue to increase in the coming years. However, a number of studies demonstrated the adverse effects of vitrification on developmental competence in oocytes and embryos.^14^, ^15^, ^16^, ^17^ Reducing developmental competence after vitrification is reportedly caused by the altered characteristics of cytoplasmic organelles and increased abnormalities of chromosomal segregation.^18^, ^19^, ^20^, ^21^ Our recent study reported that the vitrification procedure decreased the intracellular lipid content and subsequent developmental competence.^22^ In addition, the supplementation of fatty acid (FA) into the warming solution increased the intracellular lipid content and improved the developmental competence by stimulating the β‐oxidation pathway in mice and bovine. We also demonstrated that FA addition during warming improved the developmental competence of vitrified‐warmed 4‐cell stage embryos, leading to the increase of outgrowth competence in humans. However, in the previous study, the statistical power was weak due to the low sample number; therefore, to validate our previous findings, larger experiments are required. The previous study only performed embryo assessment by static microscopic observation; therefore, the impact of adding FA to warming solutions on embryonic morphokinetics is still unknown. Moreover, the efficacy of the FA addition in a clinical setting has not been evaluated. This study aimed to evaluate the efficiency of FA addition during warming on human embryonic development, including morphokinetics and clinical outcomes, by examining the development and morphokinetics of human 4‐cell stage embryos after warming with or without FA and assessing the pregnancy outcomes after single vitrified‐warmed cleavage stage embryo transfers (SVCTs).

## 2 MATERIALS AND METHODS

### 2.1 In vitro experimental study

#### 2.1.1 Embryo warming

**FIGURE 1:**
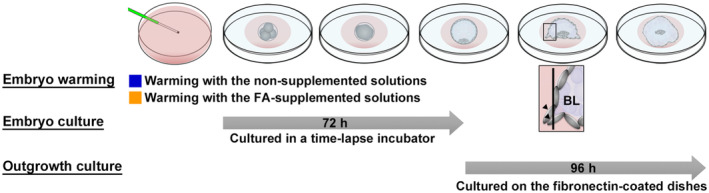
Outline of in vitro experiments. A total of 217 discarded human vitrified 4‐cell stage embryos donated for research by consenting couples were randomly allocated, to be warmed in solutions either with (FA, *n* = 111) or without FA (control, *n* = 106). The warmed embryos were cultured for 72 h in a time‐lapse incubator. Furthermore, the blastocysts produced were plated on fibronectin‐coated dishes and cultured for 96 h to assess blastocyst adhesion and outgrowth. Arrowheads indicate the trophoblast cells which are visible and expanding outward from the blastocysts. FA, fatty acid; BL, blastocyst.

This study used 217 discarded, vitrified human embryos donated for research by consenting couples who conceived babies and completed the fertility treatment (Table 1, Figure 1). These embryos were vitrified at 4‐cell stage on Day 2 for the vitrified‐warmed cleavage stage embryo transfers. The donated embryos were randomly allocated to two groups depending on the type of the warming solutions used: the FA‐supplemented solutions (VT526; Kitazato Corporation, FA group) and nonsupplemented solutions (VT506; Kitazato Corporation, control group).^22^ The warming procedures were carried out using the Cryotop method. Briefly, the tip of the Cryotop was dipped in a warming solution (thawing solution) at 37°C for 1 min, and the warmed embryos were transferred to a diluent solution. After 3 min, they were transferred to washing solution 1. After culturing for 5 min in washing solution 1, they were transferred to washing solution 2 and were cultured for 1 min.

**TABLE 1:** Developmental rates of vitrified cleavage stage embryos.

	Control	Fatty acid	*p* Value
No. of embryos used, *n*	106	111	
Age of women (years)	39.2 ± 0.3	39.0 ± 0.3	0.6583
Morphological grade*			
Grade 1, *n* (%)	30 (28.3)	30 (27.0)	0.8849
Grade 2, *n* (%)	54 (50.9)	53 (47.8)	
Grade 3, *n* (%)	20 (18.9)	25 (22.5)	
Grade 4, *n* (%)	2 (1.9)	3 (2.7)	
No. of embryos survived after warming, *n* (%)	106 (100)	111 (100)	1.0000
No. of cells degenerated after warming, *n*	0	0	1.0000
No. of 5‐cell stage embryos, *n* (%)	105 (99.1)	111 (100)	0.3050
No. of 6‐cell stage embryos, *n* (%)	104 (98.1)	111 (100)	0.1460
No. of 7‐cell stage embryos, *n* (%)	104 (98.1)	111 (100)	0.1460
No. of 8‐cell stage embryos, *n* (%)	104 (98.1)	110 (99.1)	0.5341
No. of morulae, *n* (%)	103 (97.2)	108 (97.3)	0.9543
No. of blastocysts, *n* (%)	58 (54.7)	72 (64.9)	0.1273
No. of expanded blastocysts, *n* (%)	39 (36.8)	46 (41.4)	0.4831
Morphological grade of inner cell mass			
Grade A, *n* (%)	20 (34.5)	34 (47.2)	0.1429
Grade B, *n* (%)	23 (39.7)	32 (44.4)	0.5827
Grade C, *n* (%)	15 (25.9)	6 (8.3)	0.0069
Morphological grade of trophectoderm			
Grade A, *n* (%)	18 (31.0)	35 (48.6)	0.1174
Grade B, *n* (%)	23 (39.7)	23 (31.9)	
Grade C, *n* (%)	17 (39.3)	14 (19.4)	
Morphologically good blastocysts/total blastocysts, *n* (%)	34 (58.6)	55 (76.4)	0.0302
Morphologically good blastocysts/total embryos warmed, *n* (%)	34 (32.1)	55 (49.6)	0.0089

*Note*: Values are presented as mean ± standard error of the mean (SEM) or *n* (%).

^a^ The cleavage stage embryos were morphologically evaluated according to Veeck's criteria.

#### 2.1.2 Embryo culture and annotation

The warmed embryos were immediately placed in EmbryoSlides (Vitrolife, Inc.) and were cultured individually in a 180‐μL medium (NAKA ONESTEP medium; NAKA Medical) with paraffin oil. Embryos were cultured at 37°C (gas phase: 5% O_2_, 5% CO_2_, and 90% N_2_) in an Embryoscope + time‐lapse incubator (Vitrolife) for 3 days. The embryo development was assessed using the EmbryoViewer software (Vitrolife).

Images were captured every 10 min at 11 focal planes over 5–7 days of culture. The onset time points of the following events were recorded and analyzed: start of the 5‐cell (t5), 6‐cell (t6), 7‐cell (t7), and 8‐cell (t8) stages, initiation of compaction (tSC), completion of compaction process (tM), initiation of blastulation (tSB), and formation of full (tB) and expanded (tEB) blastocysts.^23^, ^24^ The incidence of direct cleavage, in which one blastomere cleaved into three or more blastomeres,^25^ and reverse cleavage, in which two blastomeres fused into one blastomere,^26^ was monitored. The amount of fragmentation during cleavage and morula stages was annotated. During the embryo peri‐compaction period, excluded/extruded cells were identified only when they clearly displayed the presence of nuclei, as previously described.^27^ The blastocyst quality was evaluated according to the Gardner's criteria.^28^ The blastocysts graded AA, AB, BA, or BB were categorized as morphologically good blastocysts.

#### 2.1.3 Blastocyst outgrowth

To estimate the implantation capacity of blastocysts in vitro, the proportion of adhered blastocysts and the outgrowth area were examined as previously described.^29^ The culture dishes were precoated with 10 μg/mL fibronectin (Sigma−Aldrich) at 4°C overnight. Next, 20 μL of NAKA ONESTEP medium was pipetted onto each drop before adding the oil overlay. After removal of the zona pellucida using acid Tyrode's solution, the blastocysts were placed individually into the drops and cultured for 96 h in a humidified incubator (Astec) at 37°C with 5% O_2_, 5% CO_2_, and 90% N_2_ for the outgrowth culture assay. The embryos were designated as adhesion‐initiating blastocysts when the trophoblast cells were visible and expanding outward from the blastocysts (Figure 1). The outgrowth area was measured at the end of culture using the NIS‐Elements imaging software 2.0 (Nikon); the outer edge of the trophoblast was selected, and the outgrowth area was automatically calculated.

### 2.2 Retrospective cohort study

#### 2.2.1 Study patients

**FIGURE 2:**
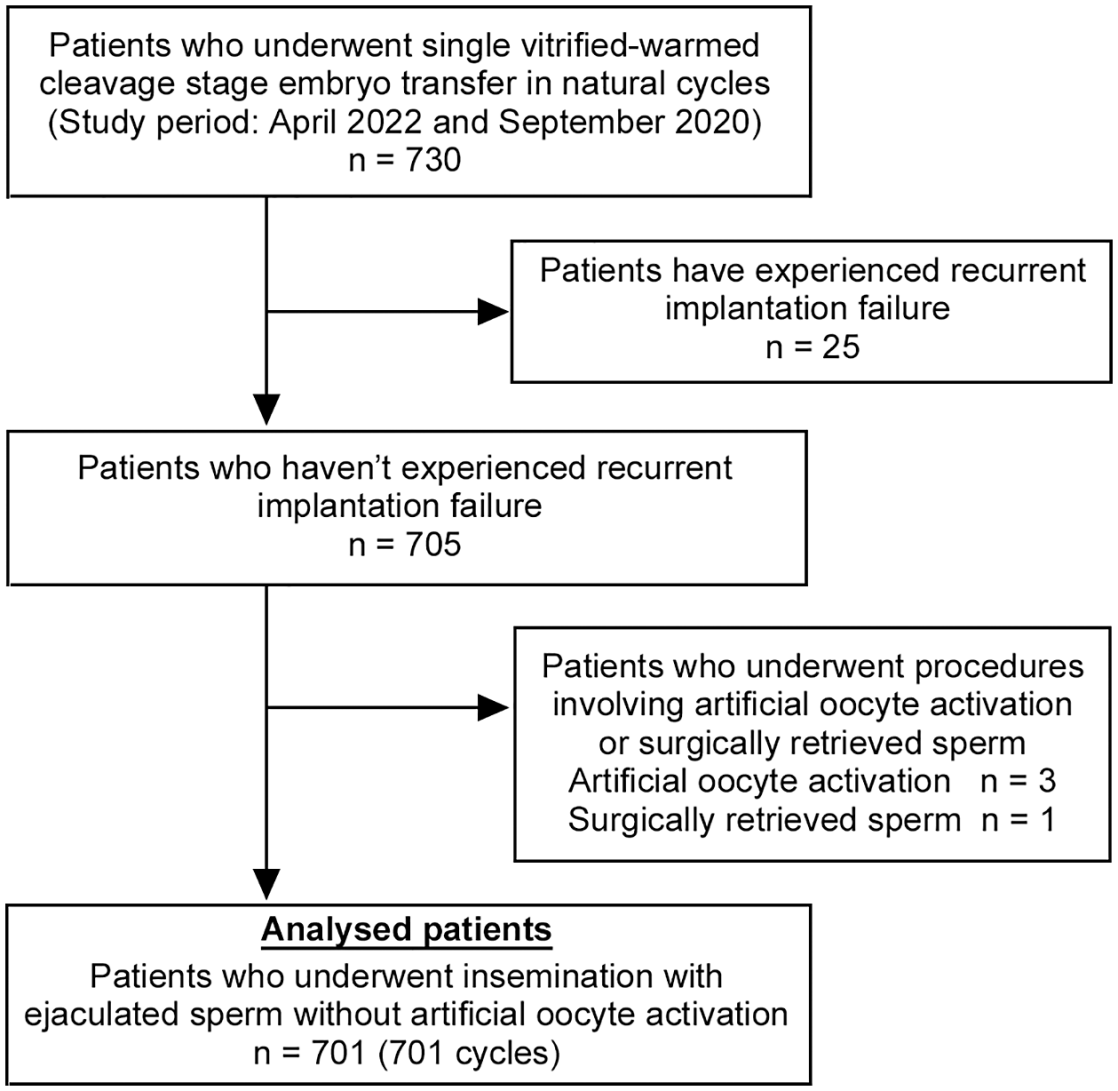
Patient selection, including inclusion and exclusion criteria. A total of 701 cycles from 701 patients were analyzed (control, *n* = 340; FA, *n* = 361). FA, fatty acid.

We reviewed the records of treatment cycles of women who underwent SVCT in natural cycles at the Kato Ladies Clinic between April 2022 and September 2022 (Figure 2). Twenty‐five patients with recurrent implantation failure (four or more unsuccessful ETs)^30^ were excluded. Patients undergoing procedures involving artificial oocyte activation (*n* = 3) or surgically retrieved sperm were excluded (*n* = 1). From April 2022 to June 2022, the vitrified embryos were warmed using FA‐nonsupplemented solutions (VT506; Kitazato Corporation, control group). From July 2022 to September 2022, the vitrified embryos were warmed using the FA‐supplemented solutions (VT526; Kitazato Corporation, FA group).

#### 2.2.2 Embryo transfer

Single vitrified‐warmed cleavage stage embryo transfers were performed as previously described.^7^ Briefly, SVCT was performed under vaginal ultrasound guidance using a specially designed soft silicone inner catheter (Kitazato Corporation); a single embryo was placed in a minimal volume in the upper part of the uterine cavity on Day 2 after ovulation in a natural cycle. Dydrogesterone (30 mg/day; Mylan EPD G.K.) was administered orally during the early luteal phase after SVCT. Implantation was defined by the serum human chorionic gonadotropin level (>20 mIU/mL) in accordance with a previous study.^31^ The clinical and ongoing pregnancy rates were defined according to the ultrasonographic observation of a gestational sac at 3 weeks after SVCT, and the observation of a fetal heartbeat was performed 5 weeks after SVCT. Early pregnancy loss and miscarriage during the first trimester were defined according to the absence of a gestational sac after implantation and the absence of a fetal heartbeat after the confirmation of a gestational sac.^32^

### 2.3 Statistical analysis

Statistical analyses were performed using the JMP software (SAS). Proportions of the data were analyzed using chi‐square test and Fisher's exact test. Continuous parameters were compared using Student's *t*‐test when normality could be accepted; otherwise, Mann–Whitney *U* test was used. Univariate logistic regression analysis was performed to identify confounders that were potentially associated with the outcomes. Multivariate logistic regression analysis was performed to adjust for bias (using the confounders) and verify the statistical significance. Adjusted odds ratios (AORs) are reported with 95% confidential intervals (CIs) for each group. The calculation of statistical power was performed using G*Power (Heinrich‐Heine‐Universität Düsseldorf). Statistical significance was set at *p* < 0.05.

## 3 RESULTS

### 3.1 In vitro experimental study

#### 3.1.1 Embryonic outcomes of the vitrified‐warmed cleavage stage embryos assessed by static observation

A total of 217 vitrified‐warmed cleavage stage embryos were used for in vitro experiments (Table 1). The age of women and morphological grade of embryos used were comparable between the control and FA groups. All embryos survived, and cell degeneration was not observed after warming in both groups. Developmental rates were comparable between the groups during the cleavage, compaction, and blastocyst stages (Table 1). However, the proportion of inner cell mass with a morphological grade of C was significantly lower in the FA group than in the control group (*p* = 0.0069). The rate of morphologically good blastocysts per obtained blastocysts was significantly higher in the FA group than in the control group (*p* = 0.0302). The rate of morphologically good blastocysts per total embryos warmed was also increased in the FA group than in the control group (*p* = 0.0089).

#### 3.1.2 Morphokinetics of the vitrified‐warmed cleavage stage embryos

The developmental timings were comparable between the groups during the cleavage, compaction, and blastocyst stages (Table 2). The incidence of abnormal cleavages in the FA group was similar to that in the control group. The amount of fragmentation at 4‐cell, 8‐cell, and morula stage was comparable between the groups. The incidence of blastomere exclusion before compaction and blastomere extrusion after compaction was also comparable between the groups. There was no difference in the proportion of compaction patterns between the groups.

**TABLE 2:** Embryonic morphokinetics and morphological alteration.

	Control	Fatty acid	*p* Value
Time interval from t5 to t6	2.0 ± 0.4	1.4 ± 0.3	0.2750
Time interval from t5 to t7	4.2 ± 0.6	3.8 ± 2.9	0.5496
Time interval from t5 to t8	8.0 ± 0.8	8.3 ± 0.7	0.7767
Time interval from t5 to tSC	30.2 ± 0.6	29.8 ± 0.7	0.6958
Time interval from t5 to tM	39.6 ± 0.7	38.1 ± 0.8	0.1622
Time interval from t5 to tSB	51.8 ± 0.6	50.4 ± 0.7	0.1174
Time interval from t5 to tB	58.5 ± 0.7	57.8 ± 0.8	0.5563
Time interval from t5 to tEB	64.8 ± 0.7	63.0 ± 0.8	0.0906
Direct cleavage at cleavage stage, *n* (%)	2 (1.9)	5 (4.5)	0.2753
Reverse cleavage at cleavage stage, *n* (%)	3 (2.8)	5 (4.5)	0.5129
Fragmentation at 4‐cell stage	11.8 ± 0.8	10.9 ± 0.6	0.3489
Fragmentation at 8‐cell stage	10.6 ± 1.0	9.5 ± 0.8	0.3573
Fragmentation at morula stage	12.2 ± 0.9	10.6 ± 1.0	0.2251
No. of blastomeres at tSC	13.8 ± 0.3	13.3 ± 0.2	0.1750
Exclusion of blastomeres before compaction			
No blastomere, *n* (%)	86 (83.5)	80 (74.1)	0.1854
1 blastomere, *n* (%)	13 (12.6)	16 (14.8)	
2 blastomeres, *n* (%)	1 (1.0)	8 (7.4)	
3 blastomeres, *n* (%)	1 (1.0)	2 (1.9)	
≥4 blastomeres, *n* (%)	2 (1.94)	2 (1.85)	
Extrusion of blastomeres after compaction			
No blastomere, *n* (%)	73 (70.9)	74 (68.5)	0.8985
1 blastomere, *n* (%)	13 (12.6)	12 (11.1)	
2 blastomeres, *n* (%)	5 (4.9)	6 (5.6)	
≥3 blastomeres, *n* (%)	12 (11.7)	16 (14.9)	
Pattern of compaction			
FCM, *n* (%)	64 (62.1)	60 (55.6)	0.4185
Exc‐PCM, *n* (%)	9 (8.7)	14 (13.0)	
Ext‐PCM, *n* (%)	22 (21.4)	20 (18.5)	
Exc/Ext‐PCM, *n* (%)	8 (7.8)	14 (13.0)	

*Note*: Start of the 5‐cell (t5), 6‐cell (t6), 7‐cell (t7), 8‐cell (t8) stages, initiation of compaction (tSC), completion of compaction process (tM), initiation of blastulation (tSB), and formation of full (tB) and expanded (tEB) blastocyst. Values are presented as mean ± SEM or *n* (%).

Abbreviations: Exc/Ext‐PCM, partially compacted morula with both excluded and extruded cells; Exc‐PCM, partially compacted morula with excluded cells; Ext‐PCM, partially compacted morula with extruded cells; FCM, fully compacted morula.

#### 3.1.3 Blastocyst outgrowth

**FIGURE 3:**
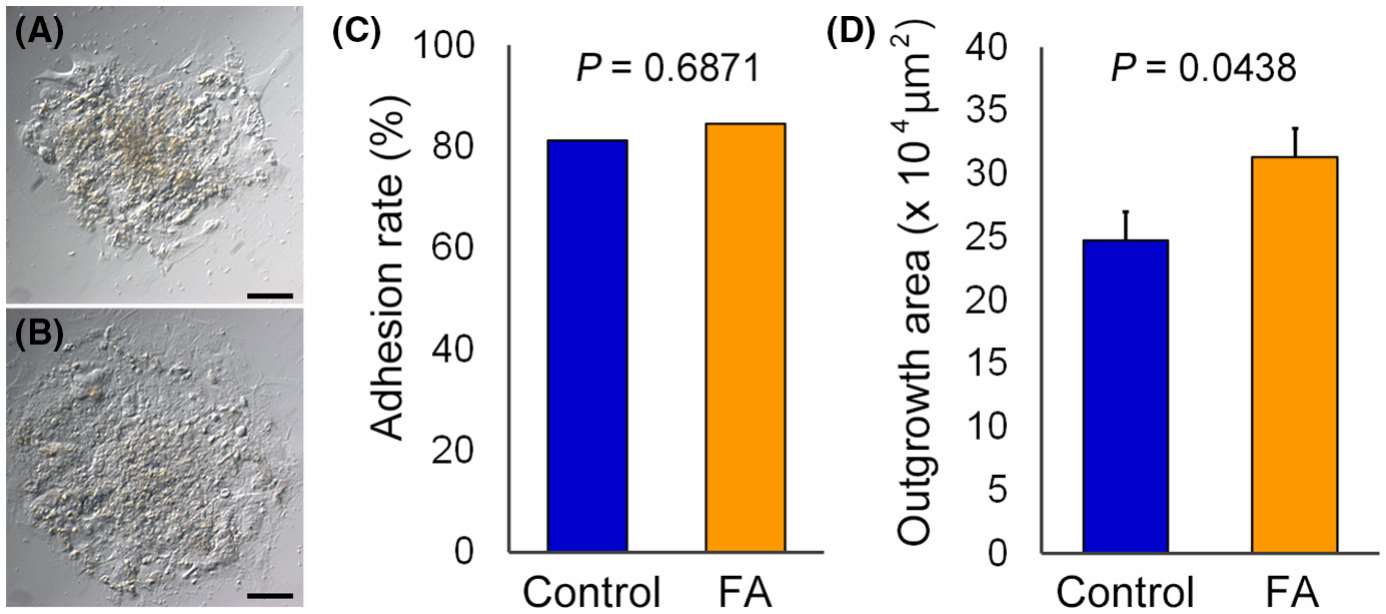
Effect of adding FAs to the warming solutions on blastocyst outgrowth. Blastocyst outgrowth after 96 h of culture in the control (A) and FA (B) groups. (C) rates of blastocyst adhesion to the fibronectin‐coated dishes, comparing the control and FA groups (control, *n* = 58; FA, *n* = 72); (D) outgrowth area after 96 h of culture, comparing the control and FA groups. FA, fatty acids. Scale bar = 100 μm. Error bars represent the standard error of the mean.

The obtained blastocysts were used for the outgrowth assay (Figure 3A,B). The adhesion rate to the fibronectin‐coated dishes at 96 h after the outgrowth culture was comparable between the groups (Figure 3C). However, the outgrowth area was significantly larger in the FA group than that in the control group (*p* = 0.0438, Figure 3D).

### 3.2 Retrospective cohort study

#### 3.2.1 Patient characteristics

The age of women and men was comparable between the control and FA groups (Table 3). The number of previous embryo transfer cycles and the proportion of infertility causes were similar between the groups.

**TABLE 3:** Patient characteristics.

	Control	Fatty acid	*p* Value
No. of patients, *n*	340	361	
Age of women (years)	37.1 ± 0.2	36.9 ± 0.2	0.4708
Age of men (years)	39.6 ± 0.3	39.5 ± 0.3	0.8978
No. of previous embryo transfer cycles	0.7 ± 0.0	0.6 ± 0.0	0.1909
Infertility cause, *n* (%)			
Ovulation factor	28 (8.2)	38 (10.5)	0.0869
Oviduct factor	3 (0.9)	8 (2.2)	
Endometriosis	12 (3.5)	15 (4.2)	
Endometrial factor	27 (7.9)	17 (4.7)	
Male factor	102 (30.0)	95 (26.3)	
Combination	62 (18.2)	51 (14.1)	
Unexplained	106 (31.2)	137 (38.0)	

*Note*: Values are presented as mean ± SEM or *n* (%).

#### 3.2.2 Pregnancy outcomes after SVCTs

The insemination method, number of blastomeres, and morphological grade of the transferred embryos were comparable between the control and FA groups (Table 4). However, the rates of implantation, clinical pregnancy, and ongoing pregnancy were higher in the FA group than those in the control group (*p* = 0.0252, *p* = 0.0223, and *p* = 0.0281, respectively). The rates of early pregnancy loss and miscarriage during the first trimester were comparable between the groups. The multivariate logistic regression analysis demonstrated that the probability of ongoing pregnancy was significantly increased in the FA group than in the control group (AOR, 1.46: 95% CI, 1.02–2.06; *p* = 0.0340; Table 5).

**TABLE 4:** Pregnancy outcomes after single vitrified‐warmed embryo transfers on Day 2.

	Control	Fatty acid	*p* Value
No. of embryo transfer cycles, *n*	340	361	
Insemination			
Conventional in vitro fertilization	134 (39.4)	144 (39.9)	0.8972
Intracytoplasmic sperm injection	206 (60.6)	217 (60.1)	
No. of blastomeres of the transferred embryos	5.4 ± 0.1	5.4 ± 0.1	0.6582
Morphological grade of the transferred embryos, *n* (%)			
Grade 1	54 (15.9)	44 (12.2)	0.2569
Grade 2	107 (31.5)	108 (29.9)	
Grade 3	179 (52.7)	209 (57.9)	
Implantation, *n* (%)	100 (29.4)	135 (37.4)	0.0252
Clinical pregnancy, *n* (%)	88 (25.9)	122 (33.8)	0.0223
Ongoing pregnancy, *n* (%)	76 (22.4)	107 (29.6)	0.0281
Early pregnancy loss, *n* (%)	12 (12.0)	13 (9.6)	0.5601
Miscarriage during the first trimester, *n* (%)	12 (13.6)	15 (12.3)	0.7745

*Note*: Values are presented as mean ± SEM or *n* (%).

**TABLE 5:** Multivariate logistic regression analysis for ongoing pregnancy rate after vitrified‐warmed embryo transfers on Day 2.

	Univariate analysis				Multivariate analysis			
	Odds ratio	95% CI	*p* Value	AUC	Adjusted odds ratio	95% CI	*p* Value	AUC
Female age	0.91	0.87–0.95	<0.0001	0.627	0.92	0.88–0.97	0.0017	0.634
Male age	0.94	0.92–0.97	0.0003	0.589	0.98	0.95–1.02	0.3663	
No. of previous ET	0.91	0.74–1.11	0.3624	0.520	—	—	—	
Morphological grade				0.545				
Grade 1	Reference	—	—		—	—	—	
Grade 2	0.88	0.52–1.47	0.6159	—	—	—	
Grade 3	0.65	0.40–1.06	0.0858	—	—	—	
Warming solution								
Control	Reference	—	—		Reference	—	—	
Fatty acid	1.46	1.04–2.06	0.0285		1.46	1.02–2.06	0.0340	

Abbreviations: AUC, area under the curve; CI, confidential interval; ET, embryo transfer.

We stratified the pregnancy outcomes by the median of women's age (median, 38 years; Table S1). Although the ongoing pregnancy rates were numerically higher in the FA group than that in the control group in both young and advanced age groups, there was no difference between the groups.

## 4 DISCUSSION

This study demonstrated that warming human cleavage stage embryos using warming solutions supplemented with FA improved the blastocyst morphology after the embryo culture for 72 h. In addition, the morphokinetics during cleavage, morula, and blastocyst stages were not altered by the FA addition. Furthermore, the outgrowth competence was increased in embryos warmed with FA. Moreover, this is the first report demonstrating that the pregnancy outcomes after SVCTs were significantly increased when the cleavage‐stage embryos were warmed with FA‐supplemented solutions.

First, we demonstrated that the blastocyst morphology and subsequent blastocyst outgrowth were significantly improved when the vitrified 4‐cell stage embryos were warmed with the FA‐supplemented solutions. We conducted a power analysis between the control and FA groups and detected a power of 83.5% for the improvement of blastocyst outgrowth; these results validate our previous findings.^22^

Fatty acids play a crucial role in energy generation, which are transferred to the mitochondria and catabolized to acetyl‐CoA, leading to adenosine triphosphate production via the mitochondrial electron transport chain.^33^, ^34^, ^35^, ^36^ Therefore, we hypothesized that the alterations of cytoplasmic fatty acid contents and mitochondrial beta‐oxidation activity in the vitrified embryos might affect morphological alteration after warming, resulting in the improvement of blastocyst quality. In this study, we also observed the morphokinetics of vitrified‐warmed 4‐cell stage embryos using time‐lapse systems. Contrary to our expectation, all the morphokinetic parameters, including the time of cell division, compaction, and blastulation, and the incidence of abnormal events, were comparable between the control and FA group. These results suggested that adding FA into warming solutions did not adversely affect the biological events during preimplantation period. However, the mechanism by which the FA supplementation into warming solutions improved embryonic qualities remains unknown; therefore, further studies are required to reveal the mechanism.

We examined the effects of FA‐supplemented warming solutions on pregnancy outcomes. The rates of implantation, clinical pregnancy, and ongoing pregnancy after SVCTs were significantly increased by adding FA into the warming solutions. From the results mentioned above, we considered that the improvement in pregnancy outcomes might be caused by the improvement in blastocyst morphology. Furthermore, we conducted a power analysis between the control and FA groups and detected a power of 98.9% for the improvement of ongoing pregnancy; therefore, the reliability of this result could be considered as high. However, to perform the subgroup analysis, the sample number is still too low to detect the statistical differences. In order to determine how effective FA‐supplemented warming solutions are for certain populations, further large cohort studies are required.

The strength of this study was its analysis of a large dataset from a single center. In addition, the endometrial preparation method, techniques of the transfer, and culture conditions were uniform. Therefore, potential bias owing to differences in the detailed conditions that potentially occur in multicenter data collection is unlikely. However, our study has limitations, such as its retrospective design. Further prospective studies are required to validate the clinical efficacy of FA‐supplemented solutions. In addition, the effectiveness of FA addition on warming human oocytes and blastocysts remained unclear. Furthermore, the maternal and perinatal outcomes after warming with FA‐supplemented solutions should be assessed in the future.

In conclusion, we demonstrated that the supplementation of FA into the warming solutions improved pregnancy outcomes after SVCTs. Combined with recent technologies, including the embryo ranking based on the time‐lapse data and artificial intelligence and PGT, the warming procedure should be optimized in each laboratory to maximize the clinical outcomes, shorten the treatment period, and reduce the patient burden.

## CONFLICT OF INTEREST STATEMENT

The authors declare no conflict of interest.

## ETHICS APPROVAL

The study was an experimental and retrospective cohort study approved by the Institutional Review Board of Kato Ladies Clinic (approval number 21‐12, 21‐23).

## HUMAN RIGHTS STATEMENTS AND INFORMED CONSENT

All procedures followed were in accordance with the ethical standards of the responsible committee on human experimentation (institutional and national) and with the Helsinki Declaration of 1964 and its later amendments. Informed consent was obtained from all patients for being included in the study.

## ANIMAL STUDIES

This article does not contain any studies with animal subjects performed by any of the authors.

## Supporting information


Table S1.
