# Supplementary material for: Fatty acid supplementation into warming solutions improves pregnancy outcomes after single vitrified‐warmed cleavage stage embryo transfers
Source: Reprod Med Biol. 2023 May 8;22(1):e12517. doi: 10.1002/rmb2.12517 (PMC10165886; doi:10.1002/rmb2.12517)
Supplement: Supplementary file 1 — Table S1. [file RMB2-22-e12517-s001.docx]

**Supporting Information**

**Supplemental Table 1. Pregnancy outcomes after vitrified-warmed embryo transfers on day 2, stratified by the age of women**

|  | Control | Fatty acid | *P* value |
| --- | --- | --- | --- |
| **Age of women: < 38 y** |  |  |  |
| No. of embryo transfer cycles, n | 167 | 181 |  |
| Implantation, n (%) | 53 (31.7) | 72 (39.8) | 0.1182 |
| Clinical pregnancy, n (%) | 48 (28.7) | 68 (37.6) | 0.0810 |
| Ongoing pregnancy, n (%) | 44 (26.4) | 62 (34.3) | 0.1093 |
| Early pregnancy loss, n (%) | 5 (9.4) | 4 (5.6) | 0.4071 |
| Miscarriage during the first trimester, n (%) | 4 (8.3) | 6 (8.8) | 0.9262 |
| **Age of women: ≥ 38 y** |  |  |  |
| No. of embryo transfer cycles, n | 173 | 180 |  |
| Implantation, n (%) | 47 (27.2) | 63 (35.0) | 0.1122 |
| Clinical pregnancy, n (%) | 40 (23.1) | 54 (30.0) | 0.1438 |
| Ongoing pregnancy, n (%) | 32 (18.5) | 45 (25.0) | 0.1392 |
| Early pregnancy loss, n (%) | 7 (14.9) | 9 (14.3) | 0.9287 |
| Miscarriage during the first trimester, n (%) | 8 (20.0) | 9 (16.7) | 0.6780 |

Abbreviations: SEM, standard error of mean.
